# Commemorative 10th Anniversary Issue of *Bioengineering*: Perspectives in Bioengineering

**DOI:** 10.3390/bioengineering11111137

**Published:** 2024-11-12

**Authors:** Anthony Guiseppi-Elie

**Affiliations:** 1 Bioelectronics, Biosensors and Biochips (C3B^®^), Department of Biomedical Engineering, Texas A&M University, College Station, TX 77843, USA; guiseppi@tamu.edu; Tel.: +1-(804)-347-9363; 2 Department of Cardiovascular Sciences, Houston Methodist Institute for Academic Medicine and Full Affiliate Member, Houston Methodist Research Institute, Houston, TX 77030, USA; 3 ABTECH Scientific, Inc., Biotechnology Research Park, 800 East Leigh Street, Richmond, VA 23219, USA

## 1. Introduction

**Figure 1:**
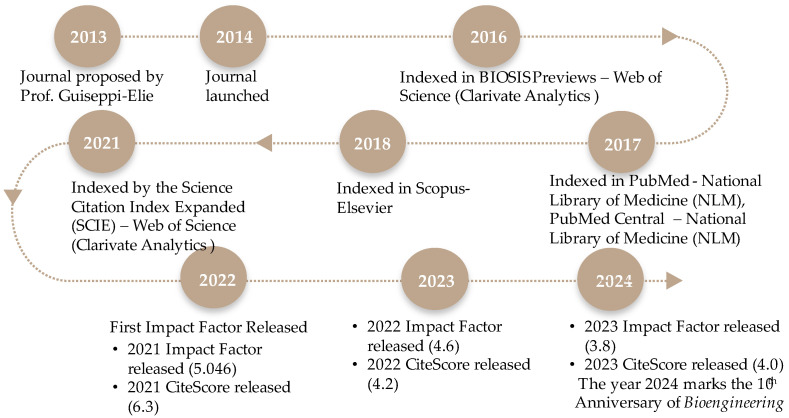
Timeline of the history of the MDPI journal *Bioengineering*.

In 2014, as the Founding Editor-in-Chief, I welcomed you to the first issue of a new open access MDPI journal, *Bioengineering* [1]. In my first editorial, I expressed my hope that *Bioengineering* would fulfill its potential to address the need for a journal in the broadly defined discipline of bioengineering that sat at the interface of the traditional disciplines of engineering in biology and biological engineering. Today, as we celebrate our 10th anniversary, I welcome you once again, this time with confidence and assurance that *Bioengineering* will help to shape the future of our discipline by (i) democratizing science and engineering scholarship through its open access platform and (ii) furthering the practice of our discipline through creative and imaginative approaches to scientific communications. These two principles are foundational to my leadership of the journal. *Bioengineering* was founded on the principle of high quality in the selection and publication of cutting-edge, high-impact research. The editorial leadership team of professional editorial managers and academic oversight editors includes distinguished members of our scholarly community of experts who oversee a rigorous peer review system. *Bioengineering* has undoubtedly fulfilled its promise to our community of scholars and authors, and we have embraced innovation through the creation of new forms of academic expression, such as the translational case report. This year, 2024, marks my 10th year as the Founding Editor-in-Chief of the MDPI open access journal *Bioengineering*. Figure 1 documents the progress of this journal from its conception in 2013, including the launch of its first issue in 2014. I am deeply grateful for the pioneering spirit and courage of my inaugural Associate Editors: Dr. Liang Luo of Huazhong University of Science and Technology (HUST), China, Dr. Rossana Madrid of the National University of Tucumán, Argentina, and Dr. Mark Blenner, then of Clemson University, USA. Together, we carved out a path to excellence within this discipline, achieving our first Impact Factor of 5.046, placing the journal at the top of the second quartile among biomedical engineering journals. Today, *Bioengineering*, with a 2023 IF of 3.8, is poised to publish ~1500 peer-reviewed journal articles across six sections, with the participation of five Section-Editors-in-Chief, two Associate Editors, a 10-member Editorial Advisory Board, and 344 Editorial Board members—a total of 362 editorial board members.

Each journal section is presided over by a dedicated and highly accomplished Section-Editor-in-Chief. Our sections are as follows:

- Biomedical Biomaterials [*SEiC*, Prof. Gary Bowlin (Google Scholar Citations 26,439| h-index = 72), University of Memphis, USA];
- Biomechanics and Sports Medicine [*SEiC,* Prof. Dr. Franz Konstantin Fuss (Google Scholar Citations—5811|h-index = 37), University of Bayreuth, Germany];
- Regenerative and Tissue Engineering [*SEiC,* Prof. Dr. E lena A. Jones (Google Scholar Citations 13,899|h-index = 50), University of Leeds, UK];
- Nano-Biotechnology [*SEiC*, Dr. Gary Chinga Carrasco (Google Scholar Citations 6944|h-index = 48), RISE PFI, Norway];
- Biosignal Processing [*SEiC,* Dr. Andrea Cataldo (According to Research Gate 2514 Citations), University of Salento, Italy];
- Biochemical Engineering [Interim *SEiC*, Prof. Dr. Liang Luo (Google Scholar Citations 3256|h-index = 29), Huazhong University of Science and Technology, China].

Our ten-member Editorial Advisory Board (EAB) [2], convened in 2024, is a group of distinguished scholars and experienced researchers nominated and selected by the journal’s EiC to advise on editorial strategy, provide guidance on the rapidly and dynamically evolving field of bioengineering, and serve as liaisons between *Bioengineering* and the professional communities that we serve. The expertise of the EAB members (with an average h-index of 96, Google Scholar 3 October 2024) spans the breadth of the journal’s sections and reflects its scope. Our Editorial Board (EB), comprising 344 active members, several of whom have been with us since our inaugural issue, serve as the cornerstone of the journal. The EB is represented by the following national distribution: 45.7% from the USA, 13.9% from China, 10.6% from Italy, 3.7% from Germany, 3.2% from the UK, and 22.9% from the rest of the world. The average h-index of the EB is 35.9. EB members are our first line of pre-screened and selected individuals for the conduct of peer-reviews, serve as Guest Editors and be surveyed for their input on broad editorial policy issues. The editorial leadership comprises an intellectually strong group. However, the United States makes up the largest proportion of the EB and hence contributes greatly to the creative content of the journal, such as peer review and the creation of Special Issues.

**Figure 2:**
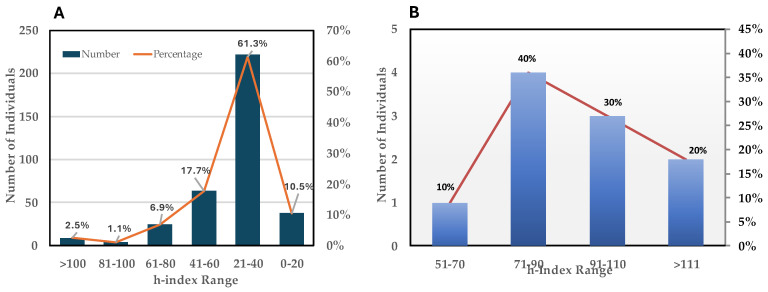
Distribution of h-index scores (Google Scholar 27 September 2024) of the *Bioengineering* Editorial Board (**A**) and Editorial Advisory Board members (**B**).

Figure 2 shows the distribution of h-indices among our Editorial Board members and Editorial Advisory Board members.

*Bioengineering* is managed by professional in-house editors, led by a Managing Editor, senior editors, and assistant editors. Together, they handle manuscripts from submission to publication, including overseeing the peer review process and production steps, while continually liaising with the SEiCs, the EiC, and the authors. Our in-house editors closely collaborate with our Editorial Board members to ensure the highest publishing standards in accordance with our publicly disclosed editorial policies. All editorial decision making resides with the SEiCs and, ultimately, with the EiC, all of whom, as shown above, have distinguished research backgrounds and hold advanced degrees and/or actively practice in the sub-fields over which they preside. The result is that *Bioengineering* boasts a turnaround time of 15.6 days between submission and first decision and, for the first half of 2024, achieved a median publication time of 39 days, with 4 days between acceptance and publication.

We deeply appreciate and value the significant contributions made by our contributing and invited authors, dedicated peer reviewers, and steadfast academic editorial board. Our editorial leadership team works in the interests of all, to ensure that all published work remains within the defined scope of the journal and to establish policies and procedures that guarantee the publication of high-quality archival articles.

Within my 2014 welcome [1], I also referenced the following key point: “…particular interest is the transdisciplinary and translational research represented by the activities within centers and institutes where the biological sciences and engineering disciplines cohabit seamlessly for a focus on solutions to global challenges in human, veterinary and ecological health, bioenergy, bioprocess and sustainability”. I promised then to keep the activity of research centers within our focus. Academic research centers represent an important but often unrecognized element of our innovation ecosystem. Academic research centers, such as the US National Science Foundation’s Engineering Research Centers (NSF-ERCs), Ireland’s Science Foundation Ireland (SFI), or New Zealand’s Science for Technological Innovation (SfTI), fund large center-based projects that connect faculty, professional researchers, and graduate and undergraduate students with their counterparts in industry and foundations to focus on challenging technical problems that have the potential to have transformative impacts on health, commerce, or the environment. A related area of focus within the scope of the journal, which I also promised to explore, was “Translational bioengineering”. Translational bioengineering emphasizes the “bench-to-bedside” and “lab-to-fab” aspects of our discipline. These are critically important in bioengineering, as a discipline, achieving its potential and fully benefiting society. In this regard, I introduced the “**translational case report**”, a type of article that provides a detailed description that highlights a direct application of scientific research in practice. It bridges the gap between basic science discoveries, engineering developments, and real-world applications, showing how laboratory research or new discoveries are translated into new and novel products, better practices, and/or improved patient outcomes.

Two articles in this commemorative issue highlight my commitment to our focus on centers and embrace the translational case report concept. The first article, that of Prof. Gordon G. Wallace of the Intelligent Polymer Research Institute (IPRI) at the University of Wollongong, NSW, Australia, recounts the trajectory of several biomedical device technologies that emerged out of scientific curiosity regarding the origins and behavior of electrical conductivity in organic polymeric materials [3]. The second is by Prof. Buddy D. Ratner of the Washington Engineered Biomaterials Center (UWEB-ERC) at the University of Washington, Seattle, WA, USA, and reports the trajectory of several biomedical device technologies that were developed out of scientific curiosity about the origins of the biomaterial contribution to the foreign body response to implanted biomedical biomaterials [4]. Both these articles emphasize the importance of a core set of driving fundamental issues, teamwork across multiple disciplines to address these issues, and the capacity to think along multiple length scales of phenomena, from molecular phenomena, through engineering and innovation, to real-world impacts.

## 2. Scope and COPE

Bioengineering is a highly interdisciplinary field that applies engineering principles to the multiple length scales of biological systems to solve problems in biology, related to the environment, industry and the practice of medicine and healthcare. The scope of *Bioengineering* is given as “Bionics and biological cybernetics: implantology; bio–abio interfaces—Bioelectronics: wearable electronics; implantable electronics; “more than Moore” electronics; bioelectronics devices—Bioprocess and biosystems engineering and applications: bioprocess design; biocatalysis; bioseparation and bioreactors; bioinformatics; bioenergy, etc.—Biomolecular, cellular and tissue engineering and applications: tissue engineering; chromosome engineering; embryo engineering; cellular, molecular and synthetic biology; metabolic engineering; bio-nanotechnology; micro/nano technologies; genetic engineering; transgenic technology—Biomedical engineering and applications: biomechatronics; biomedical electronics; biomechanics; biomaterials; biomimetics; biomedical diagnostics; biomedical therapy; biomedical devices; sensors and circuits; biomedical imaging and medical information systems; implants and regenerative medicine; neurotechnology; clinical engineering; rehabilitation engineering—Biochemical engineering and applications: metabolic pathway engineering; modeling and simulation—Translational bioengineering”.

**Figure 3:**
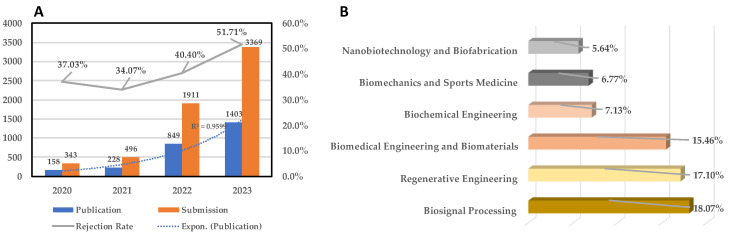
(**A**) Publications, submissions, and rejection rate (%) from 2020 to 2023. (**B**) Distribution of published articles among the various sections of the journal (data are correct as of 20 September 2024) [rejection rate = rejected papers/(rejected papers + published papers)].

*Bioengineering* has grown tremendously since its inaugural issue in 2014. It is extremely challenging to develop a journal from having ~250 to ~1400 publications annually over a four-year period. Figure 3A shows the growth in the number of publications relative to submissions and the corresponding rejection rate (%) from 2020 to 2023. This exponential growth (R^2^ = 0.959) in the number of publications is only surpassed by the growth in the number of submissions. Clearly, *Bioengineering* is fulfilling a key need. In 2022, to better manage the growth of the journal, we established several sections (referenced in the Introduction). These sections reflect the diversity of interests among the bioengineering science, technology, and innovation communities. Figure 3B shows the distribution of published articles among the various sections of the journal (data are correct as of 20 September 2024). The three dominant areas of interest, reflected in the % distribution of the published papers, are clearly Biosignal Processing (18.1%), Regenerative Engineering (17.1%), and Biomedical Engineering and Biomaterials (15.5%).

Staying within scope is difficult, and this can be exacerbated by possible inexperience and/or lack of knowledge among in-house associate and assistant editors leading them to accept any contribution with “bio” in the title as being appropriate for *Bioengineering*. Understanding the discipline of bioengineering and not confusing it with other biological disciplines, e.g., quantitative biology, clinical medicine, epidemiology, or bioinformatics, is vital. Moreover, while some of these other disciplines may contribute to our telling a comprehensive bioengineering story, it is critical that our in-house editors use the experience and expertise of our Editorial Board to check whether every submission is suitable for peer review for this specific journal. This is particularly relevant in the creation of Special Issues (SI), which must be approved by the SEiC and/or the EiC before being launched. Moreover, it is vitally important that each SI Guest Editor clearly define the scope of coverage of the SI and ensure, by working with the SEiC and/or EiC to include a minimum of four potential contributions prior to launch, that their SI is fully aligned with the scope of the journal. This is not always the case. The result is that soundly prepared articles can survive the critical scrutiny of peer review and merit publication but are otherwise outside of the defined scope of the journal. Such articles, when published, lead to what I term “scope drift”. Scope drift is not a transient, innocent singularity. Scope drift leads to an eventual redefinition of the scope of the journal. *Bioengineering*, which is presently classified as a “Biomedical Engineering” journal, is now being proposed for classification as a “Biotechnology & Applied Microbiology” journal. This is also true for all Special Issue proposals, which must be reviewed and approved by the designated SEiC and/or EiC; these are COPE (Committee on Publication Ethics) requirements. MDPI is a signatory to the Consensus Statement, supports the five working groups of United2Act Against Paper Mills, and seeks to adhere to the guidelines of COPE.

## 3. Good Academic Publication Practice (GAPP)

Put simply, predatory journals engage in predatory practices—a set of unethical or exploitative behaviors designed to maximize financial gain without the guardrails of standard editorial and peer review services commonly associated with legitimate academic publishing. What is not so simple is defining exactly what predatory practices are. And with practices being what they are, i.e., a set of behaviors derived from norms and mores in science culture, how then can we benchmark such behaviors, and against what? It is troubling when these behaviors originate from a set of organizationally held cultural values that violate widely accepted norms. These new behaviors that border on the aberrant may, in fact, arise from legitimate responses to the tectonic shifts within the science ecosystem, such as the growing worldwide number of scientists engaged in publishable research, the emergence of open access publishing vs. subscription publishing, the use of plagiarism checkers such as iThenticate or Turnitin as a first line of review, or the use of generative AI in the writing and/or review of manuscripts. There is also widespread support for the broadening of participation through the democratization of science that seeks to engage the participation of traditionally under-represented and otherwise disenfranchised groups. There is legitimate support for both sides; the challenge, as always, is where to draw the line so that these sides may be clearly identified—finding information is easy, but finding the truth is challenging. Misunderstandings can lead to high levels of discontent among the ranks of academic editors. Mass academic resignations from MDPI editorships, such as what we have witnessed for the journal *Nutrients*, represent pushback against the normalization of such behaviors [5].

For anyone who has achieved a doctorate and did not write their own dissertation using a word processor, the benchmark is the classic journal subscription model. In a recent interview, I responded to the question **“What do you think of the development of Open Access in publishing?”**. I was frank then, as I am now. “I am an advocate for open access publishing”. Open access publishing is here to stay, play its role, evolve into obsolescence, and eventually be replaced by self-publication [6]. However, until then “[open access publishing] continues to receive some scrutiny and criticism for being a double-edge sword in both perception and practice”. It is in this regard that, as a legitimate open access publication house, MDPI and *Bioengineering* can continue to improve, with the following principles:

1. No mass academic solicitation emails without signature authority and traceability to the Editor-in-Chief. That is, emails that originate from the editorial office that are intended to address editorial matters should not originate from an arbitrary email address from an un-named person.
2. Authors who request consideration for the publication of their manuscripts should use their institutional emails and be registered online with an ORCID ID. In some parts of the world, institutional emails are still quite challenging. Authors intending to facilitate and expedite communications sometimes use personal emails, such as Gmail, 123com, MSN, or Outlook.
3. We must establish a healthier and transparent working relationship between the publisher and editors. Academic editors, while maintaining their editorial autonomy and full authority in terms of accepting or rejecting papers without interference from the publisher, should share a sense of “ownership” or “stake” in the journal and hence have the opportunity to set goals, celebrate accomplishments, and voice concerns.
4. Peer review should be held to a higher standard, with the rejection of superficial or cursory efforts that do not provide substantial feedback or sufficiently scrutinize the research quality. Already, some reviewers are using generative AI to supplement/augment or replace their own reviews. An appropriate question may be, “is there a place for an AI review stage at the editorial level?”. This type of AI review, like the use of a plagiarism checker, may be employed for screening, with the results shared with the requested peer reviewer. This could have the effect of lowering the individual use of AI reviews and lead to more nuanced, insightful peer review.
5. We must be less defensive and less dismissive, displaying and demonstrating more public empathy for the legitimate concerns of the scientific community.

## 4. Telling the Story of *Bioengineering*

**Figure 4:**
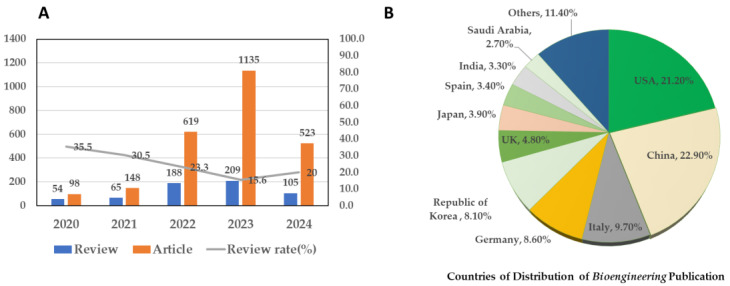
(**A**) Publication type, review vs. primary article, and their percentage (%) from 2020 to July 2024. (**B**) The distribution of published articles among the various countries of origin of contributing authors.

Against the backdrop of ongoing opportunities for collective improvement, *Bioengineering* continues to make progress as a solid Q2 journal and has a compelling story to tell. As shown in Figure 4A, the ratio of reviews to primary research articles, an inverse measure of the intellectual health of the journal, has steadily decreased and is presently ~20%. It is my hope and expectation that we will maintain this or reach a lesser amount. Figure 4B shows the distribution of published articles among the various countries of origin of our contributing authors. The USA and China continue to dominate as the principal sources of articles. However, as noted above, the USA is disproportionately represented on the EB. The consequence is that American scientists are subsidizing, through time and effort in the form of manuscript reviews, the publications of their Chinese counterparts. We shall work to nominate more Chinese scholars to the EB of *Bioengineering*. Finally, we aim to reach out and build partnerships between *Bioengineering* and professional bioengineering societies around the world. Members of these partner societies may be eligible for APC discounts. Of particular interest are professional societies in Africa, Latin America, and the Caribbean. This story will be told as, with the help of my colleague, friend, and Associate Editor, Prof. Dra. Rossana Madrid (National University of Tucumán, Argentina), we celebrate the 1st International Online Conference on Bioengineering: Bioengineering in a Generative AI World from 16 to 18 October 2024 [7].

## 5. Impact Factor

**Figure 5:**
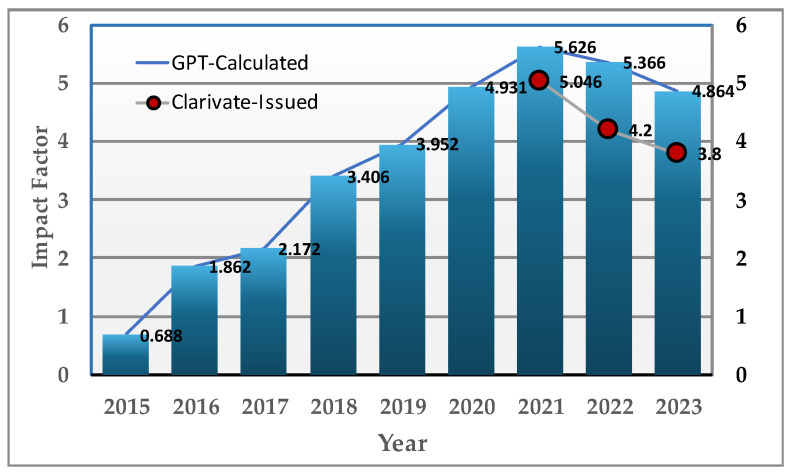
Trends in the Impact Factor of the MDPI journal *Bioengineering* from its inception to present (calculated values presented by Chat GPT).

As an Editor-in-Chief, researcher, and author, I am aware, along with others at MDPI, of the paramount importance that scholars place on a journal’s Impact Factor [8]. The Impact Factor for *Bioengineering* saw a steady increase over the early years since the journal’s launch and suffered an explainable decline in recent years. The recent downward trend in the IF may be explained by two factors: (1) the exponential growth in the number of papers published (noted earlier) favors the denominator in the IF calculation and (2) there has been a general downgrading of the IF of biomedicine journals based on Web of Science (WoB) data. Laboratory-intensive disciplines, such as biomedicine, experienced a hiatus during the COVID-19 global pandemic and its impact has now been felt. Figure 5 shows trends in the impact factor of *Bioengineering* from its inception to present. The journal saw a steady rise in its calculated Impact Factor from 2015, when it was 0.688, reaching its highest—4.9—in 2021, before undergoing a gradual decline in the following years. The journal’s Clarivate-issued Impact Factor over the years reflects this trend, with the 2021 IF being 5.046, the 2022 IF being 4.2, and the 2023 IF being 3.8, showing a steady decline following the 2021 peak. These Impact Factors reflect the journal’s increasing reputation in the field of biomedical engineering, maintaining a strong standing in the second quartile (JCR-Q2) within its academic field (*Engineering, Biomedical*) [9,10]. Despite the minor drop, *Bioengineering* remains a well-regarded journal in its field, with consistent citation rates and international participation trends that remain quite strong and positive for the future. The 2024 IF (=Citations in 2024 of articles published in 2022–2023/Total number of articles published in 2022–2023) is predicted to stabilize at ~ 3.8. Most of the citations to date originate with authors in the USA, China, and Italy. There is a need and opportunity to broaden the reach of *Bioengineering* globally.
